# Exploration of 27 plasma immune markers: a cross-sectional comparison of 64 old psychiatric inpatients having unipolar major depression and 18 non-depressed old persons

**DOI:** 10.1186/s12877-018-0836-x

**Published:** 2018-06-25

**Authors:** Torfinn Lødøen Gaarden, Knut Engedal, Jūratė Šaltytė Benth, Marianne Larsen, Bernhard Lorentzen, Tom Eirik Mollnes, Tor Magne Bjølseth, Albert Castellheim

**Affiliations:** 1 Department of Geriatric Psychiatry, Diakonhjemmet Hospital, Pastor Fangens vei 18, 0854 Oslo, Norway; 2 Norwegian Centre of Ageing and Health, and the Department of Old Age Psychiatry, Oslo, Norway; 3 Institute of Clinical Medicine, Campus Ahus, University of Oslo, Oslo, Norway; 4 Health Services Research Unit, Akershus University Hospital, Lørenskog, Norway; 5 Department of Immunology, Oslo University Hospital and K.G. Jebsen IRC, University of Oslo, Oslo, Norway; 6 Research Laboratory, Nordland Hospital Bodø and K.G. Jebsen TREC, University of Tromsø, Tromsø, Norway; 7 Centre of Molecular Inflammation Research, Norwegian University of Science and Technology, Trondheim, Norway; 8 Department of Anaesthesiology and Intensive Care, Institute of Clinical Sciences, Sahlgrenska Academy, University of Gothenburg, Gothenburg, Sweden

**Keywords:** Inflammation, Major depression, Cytokines, Immune markers, Ageing immune system, Old age

## Abstract

**Background:**

The prevalence of major depression (MD) according to population studies is the same for old (65 years and older) and younger adults. In contrast, an elevated proportion of old MD patients are hospitalized compared to younger adults with MD, indicating a need to expand the characteristics of old inpatients with MD. To illustrate this point, the association between inflammation and MD in old psychiatric inpatients is sparsely investigated even though an association between inflammation and treatment resistance among younger adults with MD has been reported. In this study, we aimed to explore the plasma concentrations of 27 immune markers in old inpatients with MD, and our purpose was to expand the understanding of inflammatory mechanisms in these patients.

**Methods:**

Prior to electroconvulsive treatment of MD, we compared 64 inpatients with unipolar MD (mean age 75.2 years) and 18 non-depressed controls (mean age 78.0 years). Symptoms characterizing MD were assessed by the Hamilton Rating Scale of Depression (HRSD)-17, and the immune markers from peripheral blood plasma were analysed using multiplex assay technology. For statistical analysis of data, we used the independent samples median test, independent samples t-test, χ^2^-test, receiver operating characteristic curve analyses, stepwise discriminant analysis, and multivariate linear regression.

**Results:**

Twenty-two immune markers representing pro- and anti-inflammatory, adaptive and trophic signalling had higher concentrations in the inpatients compared to the controls. Only the four immune markers IL-1β, IL-5, IL-10 and IL-15 had concentrations below the lower detection limit in a considerable portion (above 20%) of the patient cases. A combination of the concentration in plasma of TNF, vascular endothelial growth factor (VEGF), IL-1β, IL-7 and monocyte chemotactic protein (MCP)-1, correctly classified 98.4% of the depressed patients and 83.3% of the non-depressed controls. Plasma concentration of TNF and VEGF were associated with the HRSD-17 scores (*p* = 0.017 and 0.005, respectively).

**Conclusions:**

Our results indicate that several inflammatory mechanisms may be highly activated in old psychiatric inpatients with MD, and indicate that immune markers may contribute to a more comprehensive understanding of MD in old persons.

**Trial registration:**

NCT01559324 ClinicalTrials.gov.

**Electronic supplementary material:**

The online version of this article (10.1186/s12877-018-0836-x) contains supplementary material, which is available to authorized users.

## Background

According to population studies the prevalence of major depression (MD) in old adults (65 years and older) is reported to be about two to 5 % in the USA, Germany and Norway [1–3]. Similarly, the prevalence of MD in the general population, including young and old adults, is reported to be three to 5 % in Western Europe and Northern America [4]. Contrasting the stable prevalence of MD over time, the proportion of MD among psychiatric inpatients increases seven fold from below 30 years of age to age 70 and approaches 40% [5]. Consequently, departments of geriatric psychiatry in Norway dispose about one bed per 1000 aged 65 years and above, and non-responders to psychotherapy and anti-depressive medication treated in the primary health care are referred [6]. Thus, a better understanding of mechanisms involved in treatment resistant MD in old age may contribute to improve the quality of life and reduce the health care costs. As inflammation is associated with treatment resistance in younger adult patients with MD [7], exploring immune markers in old psychiatric inpatients with MD may contribute to expand the understanding of mechanisms that are involved.

The association between inflammation and MD in old psychiatric inpatients is sparsely examined. One study reported a 171% higher mean plasma level of interleukin (IL)-1β in 19 old inpatients compared to 21 non-depressed old controls [8]. In contrast, another study reported equal plasma concentration of IL-1β, IL-6 and tumour necrosis factor (TNF) between ten old MD psychiatric inpatients and ten non-depressed nursing home residents [9]. Additionally, several population-based, cross-sectional studies support an association between IL-6 and MD in old people [10–13] but the difference in plasma level of IL-6 between the non-depressed and those with depressed mood seems modest [12]. Accordingly, a meta-analysis including mainly young adults found a medium effect size relationship between MD and the markers IL-1 and IL-6, but suggested a dose-response relationship between MD and inflammation [14].

Ageing is associated with increased inflammation [15] and together with age related cerebral hypo-perfusion inflammation is hypothesized to be involved in development of depression in old adults [16]. Accordingly, serotonin receptor 2B (Htr2b) is reported to be upregulated during ageing in rodents [17] and Htr2b is reported to be co-localized with astrocytes and activated phagocytic microglia in peri-infarcted brain areas in humans [17]. The Astrocyte is the crucial microglia in the homeostasis of the human brain [18] and activated human astrocytes release cytokines initiating monocyte transmigration [19]. Thus, a connection between inflammation, microglia and mood regulation is suggested [20] and an understanding of these mechanisms may reveal useful biomarkers and new targets in treatment of MD in the old [20].

In our study, we decided to explore the plasma concentrations of 27 immune markers in old psychiatric inpatients with unipolar MD, because the association between inflammation and MD is sparsely investigated in old inpatients.

## Methods

### The aims of the study

In this study, we aimed to explore the plasma concentrations of 27 immune markers in old psychiatric inpatients with unipolar MD resistant to antidepressant treatment compared to a group of non-depressed old persons. Next, we aimed to ascertain whether a selection of plasma immune markers simultaneously might classify the depressed patients and the non-depressed controls. Finally, we aimed to explore the association between immune markers and the severity of symptoms characterizing MD in old persons.

### Study design

The current study is a pre-electroconvulsive treatment, exploratory study comparing old psychiatric inpatients diagnosed with unipolar MD to a non-depressed control group of old persons. The study includes a subgroup of the patients from a larger randomized controlled trial [21], registered with the identifier: NCT01559324 at the online clinical database ClinicalTrials.gov.

### Inclusion criteria

To be included, the patients were required to fulfil the criteria of the Diagnostic and Statistical Manual of Mental Disorders, Text Revision (DSM IV-TR) [22] of having a current episode of unipolar MD and having a score of at least 18 on the 17-item Hamilton Rating Scale for Depression (HRSD)-17 [23, 24]. The patient had to be between 60 to 85 years of age and had to be competent to give informed consent. All the patients were referred for electroconvulsive therapy (ECT).

### Exclusion criteria

The following were exclusion criteria; bipolar depressive disorder, Parkinson’s disease, schizophrenia, schizoaffective disorder, alcohol or substance abuse during the last three weeks, Mini Mental State Examination (MMSE) [25, 26] score of < 24 or having a diagnosis of dementia. Patients with medical conditions contradicting ECT, including all acute medical conditions and life threatening medical conditions including an advanced stage of cancer, were excluded. Likewise, patients having received ECT within the previous six months were excluded.

### Patients recruited

**Fig. 1:**
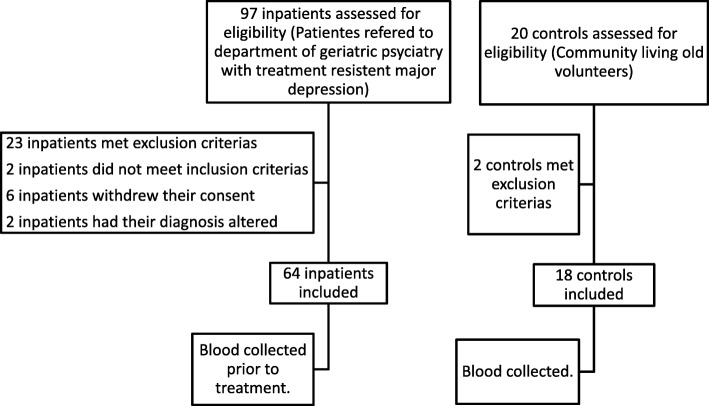
Flowchart recruitment of participants

We recruited Norwegian-speaking patients at the Diakonhjemmet Hospital, Department of Geriatric Psychiatry, a public hospital of Oslo, Norway. The department serves approximately 28,000 inhabitants aged 65 years and older. The patients were recruited during the period 1 September 2009 to 1 May 2013. Figure 1 depicts the flowchart of the recruitment process for the patients and controls. Ninety-seven patients were assessed for eligibility and 64 patients were included in the trial. Among the 33 excluded patients, 23 met exclusion criteria; two did not meet inclusion criteria; six withdrew their consent and two had their diagnosis altered.

### Diagnostic procedure

Unipolar MD was diagnosed according to the DSM-IV-TR criteria [22]. Screening for psychiatric co-morbidity was performed by a consensus between two independent senior consultants in geriatric psychiatry after a standardized clinical interview including the diagnostic structural tool Mini-international Neuropsychiatric Interview (specifically the MINI-Plus) [27, 28]. The HRSD-17 was used to rate the severity of the symptoms characterizing MD.

### Physical examination

All included patients underwent a routine physical examination with special attention to the cardiac-, respiratory- and nervous systems as a preparation for anaesthesia. The physical examination also included assessment for body mass index (BMI), blood sedimentation reaction (SR), C-reactive protein (CRP), leukocytes, haemoglobin, electrolytes, creatinine, liver-enzymes, thyroxine, HbA1c and glucose. Present and previous physical diseases affecting the immune system activity (e.g., cancer, inflammatory diseases and infections) were registered. We measured the cumulative medical burden of physical diseases using the Cumulative Illness Rating Scale for Geriatric Patients (CIRS-G) [29] (Table 1). One item regarding psychiatric disorders was excluded from the CIRS-G. The use of prescribed drugs was registered.

**Table 1:** Characteristics of the patient group and the non-depressed control group

	Patients, *N* = 64	Controls, *N* = 18	
	Mean (SD), *N* (%)	Mean (SD), *N* (%)	*P*-value
HRSD-17	23.1 (4.6)	2.7 (2.7)	< 0.001^a^
MMSE	27.7 (1.8)	28.2 (2.1)	0.432^a^
Age, years	75.2 (6.3)	78.1 (4.8)	0.083^a^
Education, years	13.6 (3.0)	13.4 (2.8)	0.805^a^
BMI	23.3 (4,6)	24.2 (4.1)	0.422^a^
CIRS-G	6.8 (3.6)	5.4 (2.4)	0.139^a^
Regular intake of drugs, number	5.2 (2.3)		
Gender, female	35 (54.7%)	12 (66.7%)	0.427^b^
	Median (Q1,Q3)		
Current depression in weeks	28 (13; 77)		

*Abbreviations*: *CIRS-G* cumulative illness rating scale for geriatric patients, *HRSD-17* Hamilton rating scale of depression, *MMSE* Mini mental state examination, *N* number, *SD* standard deviation, *BMI* body mass index

^a^Independent Samples *t*-test

^b^χ^2^-test.

### Control group

We recruited 20 non-depressed Norwegian speaking old adults from a community senior citizens centre as a control group (Table 1). Two of the controls were excluded because they had an advanced stage of cancer. This exclusion criterion also applied to the patient group. Symptoms of depression were assessed by the HRSD-17 (Table 1). We measured the cumulative medical burden of physical diseases using the CIRS-G (Table 1) from which the psychiatric item was excluded. Apart from assessing BMI, we did not perform physical examinations, and we did not register the use of psychotropic drugs in the controls.

### Blood sampling

EDTA-plasma in peripheral blood was collected between 08.00 and 10.00 a. m. from the patients and between 10.00 and 11.00 a. m. from the controls. The blood was collected from patients before interventions with ECT. The blood was immediately centrifuged at 4 °C at 3000 *x g* for 15 min and immediately stored in a local bio-bank at − 80 °C.

### Laboratory analysis

The plasma samples were analysed (in a hospital laboratory in Bodø, Norway) using a multiplex cytokine assay (Bio-Plex Human Cytokine 27-Plex Panel; Bio-Rad Laboratories Inc., Hercules, CA, USA) containing the following immune markers: IL-1β, IL-1 receptor antagonist (ra), IL-2, IL-4, IL-5, IL-6, IL-7, IL-8, IL-9, IL-10, IL-12 (p70), IL-13, IL-15, IL-17, eotaxin, basic fibroblast growth factor (bFGF), granulocyte-colony stimulating factor (G-CSF), granulocyte macrophage colony stimulating factor (GM-CSF), interferon (IFN)-γ, interferon-inducible protein (IP)-10, monocyte chemotactic protein (MCP)-1, macrophage inflammatory protein (MIP)-1α, MIP-1β, platelet derived growth factor (PDGF)-BB, regulated upon activation T cell expressed and secreted (RANTES), tumour necrosis factor (TNF)-α and vascular endothelial growth factor (VEGF) (Table 3). The samples were analysed on a Multiplex Analyzer (Bio-Rad Laboratories) according to instructions from the manufacturer. The lower detection limit was in the range of 0.24–18.84 pg/ml for the different immune markers. Values measured below the lower detection limit were extrapolated beyond standard range and values out of range were given the value 0.001 pg/ml in the statistical analysis. We classified the immune markers in the categories; pro- and anti-inflammatory, adaptive [30] and trophic based on their main signalling (Table 2).

**Table 2:** Descriptive statistics of the plasma immune markers

Immune markers		Patients, *N* = 64	Controls, *N* = 18	
Name	Signalling	Median (Q_1_, Q_3_), pg /ml	Median (Q_1_, Q_3_), pg /ml	*P*-value^f^
IL-1β	Pro-infl.	1.4 (1.1; 2.0)^a^	0.5 (0.3; 0.6)^e^	< 0.001
IL-6	Pro-infl.	6.0 (4.0; 8.0)	2.0 (0.9; 2.3)^e^	0.001
TNF	Pro-infl.	25 (14; 38)	10 (3; 13)^e^	< 0.001
IL-12	Pro-infl.	9.0 (6.0; 14.8)	0.2 (0.0; 7.5)^e^	0.030
IL-17	Pro-infl.	13 (4; 27)	0.0 (0.0; 0.0)^e^	< 0.001
IFN-γ	Pro-infl.	46 (22; 77)	25 (6; 39)	0.067
PDGFBB	Pro-infl.	17 (7; 37)	2.0 (0.0; 4.8)^e^	0.003
IL-8	Pro-infl.	10 (8; 13)	5.0 (3.8; 7.0)	< 0.001
Eotaxin	Pro-infl.	60 (47; 84)	30 (25; 43)	0.001
IP-10	Pro-infl.	574 (424; 732)	725 (577; 1000)	0.047
MCP-1	Pro-infl.	20 (17; 26)	12 (9; 14)	< 0.001
MIP-1α	Pro-infl.	3.0 (2.0; 4.0)	2.0 (0.9; 3.0)^e^	0.052
MIP-1β	Pro-infl.	54 (46; 65)	42 (35; 52)	0.022
RANTES	Pro-infl.	1116 (633; 2711)	571 (347; 850)	0.016
IL-1ra	Anti-infl.	67 (42; 103)	18 (0; 29)^e^	0.005
IL-10	Anti-infl.	0.0 (0.0; 1.1)^c^	0.0 (0.0; 0.6)^e^	0.839
IL-2	Adaptive	5.0 (2.3; 8.0)	0.1 (0.0; 2.3)^e^	0.001
IL-4	Adaptive	2.0 (1.0; 2.0)	1.0 (1.8; 1.0)	0.208
IL-5	Adaptive	1.2 (1.0; 1.5)^b^	0.9 (0.8; 1.1)^e^	< 0.005
IL-7	Adaptive	6.0 (4.3; 7.8)	1.3 (0.1; 2.3)^e^	< 0.001
IL-9	Adaptive	12 (8; 15)	3.5 (0.4; 8.3)^e^	0.003
IL-13	Adaptive	4.5 (3.0; 8.0)	2.0 (1.3; 3.0)^e^	0.002
IL-15	Adaptive	2.5 (0.0; 4.8)^d^	0.0 (0.0; 0.0)^e^	< 0.001
GMCSF	Trophic	13 (9; 18)	6.0 (0.7; 10.0)	< 0.001
GCSF	Trophic	25. (16; 35)	0.0 (0.0; 12.3)^e^	< 0.001
bFGF	Trophic	26 (15; 36)	0.0 (0.0; 15.5)^e^	0.001
VEGF	Trophic	15 (9; 23)	0.0 (0.0; 1.5)^e^	< 0.001

*Abbreviations*: *Anti-infl.* Anti-inflammatory, *bFGF* basic fibroblast growth factor, *G-CSF* granulocyte-colony stimulating factor, *GM-CSF* granulocyte macrophage colony stimulating factor, *IFN* interferon, *IL* interleukin, *IP-10* interferon-inducible protein, *MCP-1* monocyte chemotactic protein, *MIP* macrophage inflammatory protein, *ml* millilitre, *N* number, *PDGF-BB* platelet derived growth factor-BB, *pg* picogram, *Pro-infl.* Pro-inflammatory, *Q* quartile, *RANTES* regulated upon activation T cell expressed and secreted, *TNF* tumour necrosis factor, *VEGF* vascular endothelial growth factor

^a^ The concentration values were extrapolated beyond standard range in 49 cases.

^b^ The concentration values were extrapolated beyond standard range in 50 cases.

^c^ The concentration values were extrapolated beyond standard range in 23 cases and the concentration values were out of range in 35 cases.

^d^ The concentration values were extrapolated beyond standard range in three cases and the concentration value were out of range in 22 cases.

^e^ The concentration values were out of range or extrapolated beyond standard range in more than 20% of the cases.

^f^ Independent samples median test.

### Statistical analysis

The demographic and clinical characteristics were presented as group means and standard deviations (SDs) or frequencies and percentages. We compared group characteristics of patients and controls, as well as patients with and without physical diseases affecting the immune system activity, using independent samples *t*-test and χ^2^-test. The immune markers were non-normally distributed, and therefore we described them using medians and first and third quartiles. We conducted group comparisons between the patients with and without physical diseases affecting the immune system activity as well as between the patients and the controls using the independent samples median test. As the distribution of most immune markers was highly skewed, we used the LN-transformed values in all further analysis. We assessed correlations among immune markers using Pearson’s correlation coefficients.

Receiver operating characteristic (ROC) curve analysis was performed on 27 immune markers to assess their ability to classify depressed patients and non-depressed controls. We selected the immune markers with an area under the ROC curve (AUC) of at least 0.85 and a specificity of at least 0.85 for a stepwise discriminant analysis (DA), as we aimed at defining a selection of a few factors that could simultaneously classify depressed patients and non-depressed controls. We adopted a cut-off of 0.85 to ensure that only immune markers with good classification ability would be included in the DA. The immune markers chosen by a stepwise DA were further explored in bivariate and multivariate linear regression models for a continuous HRSD-17 score. We entered the interaction terms between each immune marker and the variable identifying patients and controls into the regression model. A significant interaction implies that the immune marker is associated to the HRSD-17 score differently among the depressed patients and the non-depressed controls. All non-significant interactions were excluded. Finally, we adjusted the multivariate regression model for gender, age, CIRS-G scores and BMI.

The IBM SPSS Statistics 22 software and STATA v.12 were used for statistical analyses. All tests were two-sided, and we deemed the results with *p*-values below 0.05 to be statistically significant.

## Results

### Concentration of plasma immune markers in the patients versus the controls

We found higher concentrations of 22 immune markers (IL-1β, IL-1ra, IL-2, IL-5, IL-6, IL-7, IL-8, IL-9, IL-12, IL-13, IL-15, IL-17, eotaxin, bFGF, G-CSF, GM-CSF, MCP-1, MIP-1β, PDGF-BB, RANTES, TNF and VEGF) and lower concentration of IP-10 in the patients compared to the controls (Table 2). On the other hand, the concentrations of IL-4, IL-10, IFN-γ and MIP-1α were not significantly different between the two groups (Table 2).

Only four immune markers (IL-1β, IL-5, IL-10 and IL-15) had concentrations below the lower detection limit in a considerable portion (77, 78, 91 and 39% respectively) of the patient cases. The remaining 23 immune markers had concentrations above the lover detection limits in 81–100% of the patient cases. In contrast, 18 immune markers (IL-1β, IL-1ra, IL-2, IL-5, IL-6, IL-7, IL-9, IL-10, IL-12, IL-13, IL-15, IL-17, bFGF, G-CSF, MIP-1α, PDGF-BB, TNF and VEGF) had concentrations below the lower detection limit in more than 20% of the cases in the controls (Table 2).

Gender, age, physical health, BMI, cognition and education were not significantly different between the groups (Table 1).

### The impact of physical disorders on the immune markers within the patient group

Within the patient group, 37 patients with reported physical diseases affecting the immune activity had worse but not significantly different (*p* = 0.074) physical health as measured with CIRS_G (mean CIRS-G = 7.5) compared to 27 patients without reported physical diseases affecting the immune system activity (mean CIRS-G = 5.9) (Additional file 1: Table S1). Of the 27 assessed immune markers, only the plasma concentration of IL-5, IL-8 and VEGF were significantly higher in the patients with reported physical diseases affecting the immune system activity (*p* = 0.015, 0.020 and 0.016, respectively) compared to the patients without physical diseases affecting the immune system activity. Because of the similarity in concentration of immune markers between these two groups of patients, we treated the patients as one group.

### Classification of patients and controls by a panel of plasma immune markers

According to the ROC curve analyses (Table 3), nine markers (IL-1β, IL-1ra, IL-7, IL-8, IL-17, GM-CSF, MCP-1, TNF and VEGF) discriminated well between the patients and the controls as defined by the area under the ROC curve (AUC) of at least 0.85, and with a specificity of at least 0.85. The concentrations of all immune markers correlated on a range of weakly to strongly (data not shown).

**Table 3:** The receiver operating characteristic curve analyses of plasma immune markers

Immune markers	AUC ^a^ CI (95%)	Sensitivity	Specificity
IL-1β	0.97 (0.93; 1.00)	0.94	0.95
IL-1ra	0.86 (0.74; 0.99)	0.89	0.85
IL-2	0.84 (0.72; 0.96)	0.81	0.75
IL-4	0.78 (0.68; 0.87)	0.63	0.90
IL-5	0.80 (0.70; 0.90)	0.81	0.75
IL-6	0.86 (0.72; 1.00)	0.94	0.80
IL-7	0.94 (0.85; 1.00)	0.94	0.90
IL-8	0.93 (0.86; 0.99)	0.84	0.85
IL-9	0.86 (0.74; 0.98)	0.83	0.70
IL-10	0.52 (0.38; 066)	0.44	0.65
IL-12	0.84 (0.70; 0.97)	0.92	0.75
IL-13	0.81 (0.69; 0.92)	0.70	0.90
IL-15	0.81 (0.74; 0.88)	0.63	1.00
IL-17	0.89 (0.79; 1.00)	0.94	0.85
Eotaxin	0.88 (0.79; 0.97)	0.80	0.80
bFGF	0.86 (0.77; 0.96)	1.00	0.60
GCSF	0.93 (0.85; 1.00)	0.98	0.75
GMCSF	0.86 (0.78; 0.95)	0.56	1.00
IFN-γ	0.71 (0.58; 0.83)	0.59	0.80
IP-10	0.69 (0.56; 0.83)	0.02	1.00
MCP-1	0.90 (0.84; 0.97)	0.81	0.90
MIP-1α	0.72 (0.58; 0.87)	0.98	0.45
MIP-1β	0.75 (0.60; 0.89)	0.78	0.65
PDGFBB	0.83 (0.70; 0.96)	0.86	0.75
RANTES	0.71 (0.58; 0.84)	0.64	0.75
TNF	0.88 (0.80; 0.96)	0.75	0.90
VEGF	0.98 (0.95; 1.00)	0.92	0.95

*Abbreviations*: *AUC* area under curve, *bFGF* basic fibroblast growth factor, *CI* confidence interval, *G-CSF* granulocyte-colony stimulating factor, *GM-CSF* granulocyte macrophage colony stimulating factor, *IFN* interferon, *IL* interleukin, *IP-10* interferon-inducible protein, *MCP-1* monocyte chemotactic protein, *MIP* macrophage inflammatory protein, *PDGF-BB* platelet derived growth factor-BB, *RANTES* regulated upon activation T cell expressed and secreted, *TNF* tumour necrosis factor, *VEGF* vascular endothelial growth factor

^a^ AUC: Area under the receiver operating characteristic (ROC) curve

**Fig. 2:**
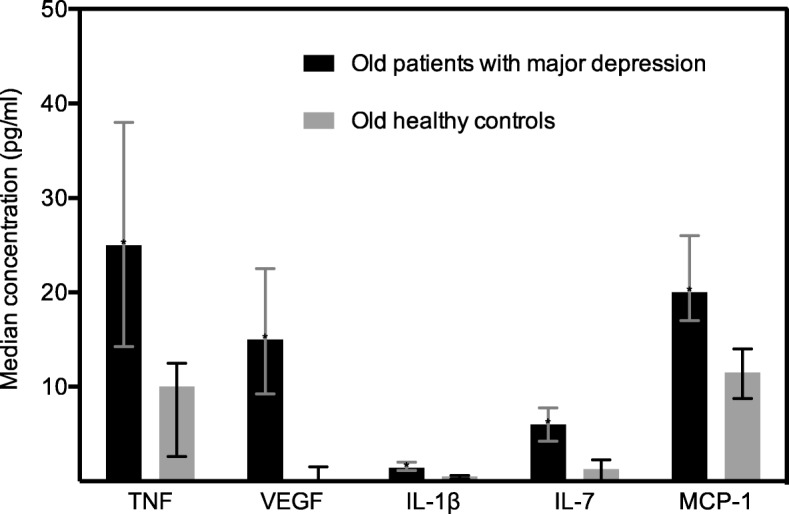
Plasma immune markers associated with the severity of symptoms characterizing unipolar major depression. The median concentration of the plasma immune markers in old patients with unipolar major depression and old non-depressed controls are illustrated by the boxes. First and third quartiles are illustrated by the error bars. Abbreviations: pg/ml, picogram/millilitre; TNF, tumour necrosis factor; VEGF, vascular endothelial growth factor; IL, interleukin; MCP-1, monocyte chemotactic protein

A step-wise discriminant analysis on the nine immune markers identified by ROC analysis suggested that the five markers, VEGF, IL-7, MCP-1, TNF and IL-1β (Fig. 2) are sufficient to classify the patients and the controls without loss of discriminatory power. The five markers correctly classify 98.4% of the patients and 83.3% of the controls, implying a correct classification rate of 95.1%.

### The association between immune markers and the severity of symptoms characterizing MD

No significant interactions were found in bivariate linear regression models, implying that there are no differences between the patients and the controls regarding the positive association between the HRSD-17 score and each of the five immune markers. In the multivariate linear regression model IL-7, TNF and VEGF were significantly associated with the HRSD-17 score. After adjusting for age, gender, CIRS-G and BMI; only TNF and VEGF remained significantly associated with the HRSD-17 score (Table 4). This model explained 53.3% of the total variance of the HRSD-17 score.

**Table 4:** Linear regression models, immune markers associated with the Hamilton rating scale of depression

	Bivariate linear reg. Models		Linear regression model assessing five immune markers simultaneously with HRSD-17			
			Unadjusted		Adjusted^a^	
Immune markers	Reg. coef. (SE)	*p*-value	Reg. coef. (SE)	*p*-value	Reg. coef. (SE)	*p*-value
IL-1β.^b^	7.2 (1.1)	< 0.001	3.1 (2.2)	0.157	3.7 (2.2)	0.101
IL-7.^b^	3.2 (0.5)	< 0.001	1.7 (0.7)	0.017	1.5 (0.7)	0.059
MCP-1.^b^	8.9 (1.9)	< 0.001	2.4 (1.9)	0.212	3.0 (2.0)	0.146
TNF.^b^	2.6 (0.7)	< 0.001	−2.5 (1.0)	0.015	−2.5 (1.0)	0.017
VEGF.^b^	1.8 (0.2)	< 0.001	1.2 (0.4)	0.002	1.1 (0.4)	0.005

*Abbreviations*: *HRSD-17* Hamilton rating scale of depression, *CIRS-G* cumulative illness rating scale for geriatric patients, *BMI* body mass index, *Reg* regression, *coef* coefficient, *SE* standard error, *IL* interleukin, *MCP-1* monocyte chemotactic protein, *TNF* tumour necrosis factor, *VEGF* vascular endothelial growth factor

^a^Adjusted for age, gender, CIRS-G (continuous) and BMI

^b^*LN transformed*.

## Discussion

### Effect size of the relationship between inflammation and MD

Our finding showing a higher concentration of a variety of immune markers representing pro- and anti-inflammatory, adaptive and trophic signalling indicates that several immune mechanisms may be involved in MD in old psychiatric inpatients. Additionally, our results indicate that the immune system may be highly activated in old psychiatric inpatients with MD, and this is consistent with a meta-analysis suggesting a dose-response relationship between inflammation and MD [14]. Further, the meta-analysis based mainly on adult outpatients reported medium effect size in the relationship between inflammation and MD [14]. The explorative approach of our study with a study population capable of detecting only large effect sizes between inflammation and MD, excluded reliable hypothesis testing. Therefore, identifying a more precise effect size in the relationship between inflammation and MD in old psychiatric inpatients requires further studies. However, our sample of old depressed inpatients is still the largest one in which study focused on the relationship between inflammation and MD. Thus, our study may contribute to better power calculations in the planning of future studies and testing of our hypothesis.

### Low plasma concentrations of immune markers

We found low plasma concentrations of IL-1β, IL-5, IL-10 and IL-15 in the patients and the controls, which is a finding in line with a previous study reporting low serum concentration of IL-10 in adult depressed out-patients and non-depressed controls [31]. The low concentrations make our comparisons of these four immune markers between the two groups less reliable; further exploration of these markers in old psychiatric inpatients with MD may require more sensitive laboratory methods than the multiplex analyses [32]. Furthermore, 14 immune markers had plasma concentrations below the lower detection limit in more than 20% of the cases in the controls. However, values out of range were given the value 0.001 pg/ml and the concentrations were compared using medians to limit the risk of overestimating the differences between the groups.

### High levels of trophic cytokines in the patient group compared to the controls

The trophic cytokines bFGF, G-CSF, GM-CSF and VEGF were higher in the patients compared to the controls. That is the opposite of what may be expected, as the more widely studied brain derived neurotrophic factor (BDNF) seems to be lower in untreated patients with MD compared to healthy controls [33]. On the other hand, higher level of VEGF in the patients with MD compared to the controls is a finding consistent with reports in previous studies [34]. Finally, pro-inflammatory cytokines like IL-1, TNF and INF-γ may induce GM-CSF, and could at least explain the high concentration of GM-CSF [35].

### Classification of patients and controls by a selection of plasma immune markers

Applying step-wise DA on a large number of immune markers implies some risk of detecting false effects and failing to select the best subset of markers able to describe differences between the two groups. To reduce the latter problem, we performed ROC curve analysis prior to DA to identify the most promising markers that could separate the patients from the controls.

Our explorative statistical analyses elucidating immune markers that may classify old persons correctly into the depressed and the non-depressed group suggested that only five markers (VEGF, IL-7, MCP-1, TNF and IL-1β) simultaneously classified about 95% of the persons correctly. To our knowledge, this has not been reported previously. However, the analyses imply a risk for Type 1 error and were not based on prior knowledge about the immune markers’ ability to separate depressed patients from non-depressed controls. Therefore, the ability of these five immune markers to separate depressed from non-depressed should be replicated in a new population.

### The association between immune markers and the severity of symptoms characterizing MD

We found a significant association between the severity of depression rated by the HRSD-17 and the levels of the three immune markers VEGF, IL-7, and TNF in the multivariate regression model. After adjusting the associations for age, gender and CIRS, only VEGF and TNF remained significantly associated to the HRSD-17 score. In contrast to the bivariate linear regression model, the multivariate regression model suggested a negative association between TNF and HRSD-17 (Table 4), which is likely due to over-adjustment in the model caused by strong correlations among the immune markers (Additional file 2: Table S2). Our finding deviates from that of Thomas et al. [8] who found a correlation between the level of IL-1β and the severity of the depression in old persons. Additionally, our finding contrasts with the results of Brambilla et al. [9], who found no correlation at all between the severity of the depression and the levels of inflammatory cytokines in old persons. However, Brambilla et al. [9] included only ten old patients with MD and consequently may not have had enough statistical power to detect putative group differences.

A small number of patients in this and previous studies, as well as differences in the study populations, may have contributed to the conflicting results.

### Limitations regarding the study groups

Limited inclusion and exclusion criteria were defined for the controls in our study and did not ensure an optimal match of patients and controls. However, the groups did not differ significantly regarding gender, age, physical health, BMI, cognition and education, and this indicates an acceptable match between the groups. Still, we do not know if the groups differed in health behaviours, personality and emotional loneliness (Additional file 3: Table S3). Nevertheless, according to Mottus et al. [36] health behaviours such as smoking, alcohol intake and physical activity did not significantly affect inflammation in old. However, personality had a minor impact on inflammation in old persons [36]. Likewise, loneliness seems to have limited impact on inflammation in adults [37]. Thus, potential differences in health behaviour, personality and emotional loneliness between the patients and the controls in our study may potentially alter our results to a slight extent.

The controls were not examined in the same way as the patients. Physical diseases were assessed by self-reporting only; medication was not assessed, and MD was excluded by using the HRSD-17, not by a psychiatric interview. Consequently, we may have missed pro-inflammatory medical conditions and we may have included controls with MD in remission with ongoing anti-depressive medication leading to an underestimation of the differences between the groups. However, because physical diseases such as heart disease and cancer seem to have a small impact on the levels of immune markers [38–42] compared to MD, a possible underestimation of the difference between the groups is probably small. This is also in line with our results where only three of 27 immune markers had significantly higher concentrations in patients with physical diseases affecting the immune system activity compared to the patients without. Likewise, if we accidentally included MD outpatients in remission and groups such as controls, this would probably not have altered our results because the levels of cytokines seem to be equal between outpatients treated with anti-depressive medication and healthy controls [43].

Our patient group represents old MD psychiatric inpatients that have not responded to medication and psychotherapy as outpatients, therefore our results should not be projected to younger adults or outpatients.

### Limitations in the study design and data collections

The explorative cross-sectional design of this study excludes reliable testing of hypotheses and determination of the temporality in the associations demonstrated between immune markers and symptoms characterizing MD. Additionally, exploring a wide range of immune markers increases the risk of identifying false effects (Type I error) and the limited number of participants implies that only large effects sizes may be demonstrated (Type II error). Further, the accuracy of the data may be affected by the self-reporting nature of data sampling in the control group. Lastly, the plasma concentrations of several immune markers in the control group were below the lower-detection level, which may also influence the accuracy of comparison between the patient group and the controls.

## Conclusion

Our results indicate that several inflammatory mechanisms may be highly activated in old psychiatric inpatients with MD, and indicate that immune markers may contribute to a more comprehensive understanding of MD in old persons.

## Additional files

Additional file 1: **Table S1.** Characteristics of the patients. The variables; HRSD-17, age, BMI, CIRS-G, gender, number of drugs and current depression in weeks are compared between patients with and without physical diseases affecting the immune system activity. (DOCX 15 kb)

Additional file 2: **Table S2.** Correlation among the immune markers and the HRSD-17. Correlation among IL-1β, IL-7, MCP-1, TNF, VEGF and the HRSD-17. (DOCX 17 kb)

Additional file 3: **Table S3.** Dataset supporting. Data on all characteristics and the concentrations of all 27 immune markers of 64 patients and 18 controls. (XLSX 25 kb)

## Abbreviations

AUC: Area under curve
bFGF: Basic fibroblast growth factor
BMI: Body mass index
CIRS-G: Cumulative illness rating scale for geriatric patients
CRP: C-reactive protein
DA: Discriminant analysis
ECT: Electroconvulsive therapy
G-CSF: Granulocyte-colony stimulating factor
GM-CSF: Granulocyte macrophage colony stimulating factor
HRSD-17: Hamilton rating scale of depression
IFN: Interferon
IL: Interleukin
IP-10: Interferon-inducible protein
MCP-1: monocyte chemotactic protein
MD: Major depression
MIP: Macrophage inflammatory protein
ml: Millilitre
MMSE: Mini mental state examination
PDGF-BB: Platelet derived growth factor-BB
pg: Picogram
PIC: Pro-inflammatory cytokine
RANTES: Regulated upon activation T cell expressed and secreted
ROC: Receiver operating characteristic
SD: Standard deviation
SR: Sedimentation reaction
TNF: Tumour necrosis factor
VEGF: Vascular endothelial growth factor
