# Supplementary material for: Exploration of 27 plasma immune markers: a cross-sectional comparison of 64 old psychiatric inpatients having unipolar major depression and 18 non-depressed old persons
Source: BMC Geriatr. 2018 Jun 25;18:149. doi: 10.1186/s12877-018-0836-x (PMC6020236; doi:10.1186/s12877-018-0836-x)
Supplement: Supplementary file 1 — Table S1. Characteristics of the patients. The variables; HRSD-17, age, BMI, CIRS-G, gender, number of drugs and current depression in weeks are compared between patients with and without physical diseases affecting the immune system activity. (DOCX 15 kb) [file 12877_2018_836_MOESM1_ESM.docx]

Table S1. Characteristics of the patients.

|  | Patients with physical diseases ^a^, N = 37 |  | Patients without physical diseases ^b^, N = 27 |  |
| --- | --- | --- | --- | --- |
|  | Mean (SD), N (%) |  | Mean (SD), N (%) | P-value |
| HRSD-17 | 22.0 (4.3) |  | 24.6 (4.6) | **0.025** ^c^ |
| Age, years | 76.0 (6.2) |  | 74.2 (6.4) | 0.243 ^c^ |
| BMI | 23.6 (4.9) |  | 22.8 (4.3) | 0.519 ^c^ |
| CIRS-G | 7.5 (3.9) |  | 5.9 (3.1) | 0.074 ^c^ |
| Female | 19 (51.4 %) |  | 16 (59.3 %) | 0.615 ^d^ |
| Drugs, N. | 5.8 (2.4) |  | 4.37 (1.6) | **0.009** ^c^ |
|  |  |  |  |  |
|  | Median (Q1, Q3) |  | Median (Q1, Q3) |  |
| Depr. ^e^, W. | 24 (12; 68) |  | 49 (16; 104) | 0.311 ^f^ |

a) Patients with physical diseases affecting the immune system activity.

b) Patients without physical diseases affecting the immune system activity.

c) Independent samples *t*-test.

d) χ^2^-test.

e) Current depression in weeks.

f) Independent samples median test.

Abbreviations: CIRS-G, Cumulative Illness Rating Scale for Geriatric Patients; HRSD-17, Hamilton Rating Scale of Depression; N, number; SD, standard deviation; Q, quartiles; BMI, body mass index; W., weeks.
