# Supplementary material for: Exploration of 27 plasma immune markers: a cross-sectional comparison of 64 old psychiatric inpatients having unipolar major depression and 18 non-depressed old persons
Source: BMC Geriatr. 2018 Jun 25;18:149. doi: 10.1186/s12877-018-0836-x (PMC6020236; doi:10.1186/s12877-018-0836-x)
Supplement: Supplementary file 2 — Table S2. Correlation among the immune markers and the HRSD-17. Correlation among IL-1β, IL-7, MCP-1, TNF, VEGF and the HRSD-17. (DOCX 17 kb) [file 12877_2018_836_MOESM2_ESM.docx]

Table S2. Correlation among the immune markers and the HRSD-17

| Immune markers | IL-1β | IL-7 | MCP-1 | TNF | VEGF |
| --- | --- | --- | --- | --- | --- |
| HRSD-17 | 0.58 | 0.56 | 0.46 | 0.38 | 0.67 |
| IL-1β |  | 0.70 | 0.58 | 0.81 | 0.78 |
| IL-7 |  |  | 0.47 | 0.71 | 0.66 |
| MCP-1 |  |  |  | 0.50 | 0.53 |
| TNF |  |  |  |  | 0.62 |
| VEGF |  |  |  |  |  |

Abbreviations: HRSD-17, Hamilton Rating Scale of Depression; IL, interleukin; MCP-1, monocyte chemotactic protein; TNF, tumour necrosis factor; VEGF, vascular endothelial growth factor.
